# Clinical photography in a high risk foot clinic: a quality audit

**DOI:** 10.1186/1757-1146-4-S1-P58

**Published:** 2011-05-20

**Authors:** Jessica M White, Hugh G Dickson, Erika N Koo, Gentry S Winters, Marion G Harpur, John Widdup, Namson S Lau

**Affiliations:** 1 Department of Podiatry, Liverpool Hospital, Locked Bag 7103, Liverpool, NSW, 2170, Australia; 2 Department of Ambulatory Care, Liverpool Hospital, Locked Bag 7103, Liverpool, NSW, 2170, Australia

## Background

The High Risk Foot Clinic at Liverpool Hospital has employed a standardised system for monitoring the progress of ulcers of the feet since January 2010. A form is completed for each patient with fields for identification, clinical photograph, and clinical details. The forms are reviewed for all patients under current treatment at a weekly meeting. We reviewed the foot ulcer progress charts, examining in particular the quality of the clinical photographs.

## Method

A manual review of the charts in use from January 2010 until 30 September 2010 was performed by a single reviewer. Data was collected on number of charts per patient, presence of a clinical photograph, quality of the photograph (focus, lighting and distance), completion of clinical details, and University of Texas wound classification. Data was entered into an Excel^©^ spreadsheet.

## Results

The review included 732 charts. Of these, 731 had a photograph, and 623 (85%) were of good quality. The most commonly completed clinical detail was “probe to bone - yes/no” (99%), followed by “antibiotic - yes/no” (97%), “offloading device” (81%), and Texas Wound Classification (76%). The range of charts per person was 1-23.

## Conclusions

The completion of data on our form appeared to follow the form design, with the frequency of items completed echoing the position of the item on the form. Anecdotally, our group has found clinical photographs to be of great assistance in monitoring our patients, especially when discussing the need for biopsy and for additional off-loading. However, the audit indicated that opportunities for improvement exist. The most common error in photography in our group is failure to use the macro setting to allow close-up images. Fortunately, digital photographs are easy to repeat.

